# Should Medical Assistance in Dying Be Extended to Incompetent Patients With Dementia? Research Protocol of a Survey Among Four Groups of Stakeholders From Quebec, Canada

**DOI:** 10.2196/resprot.8118

**Published:** 2017-11-13

**Authors:** Gina Bravo, Claudie Rodrigue, Vincent Thériault, Marcel Arcand, Jocelyn Downie, Marie-France Dubois, Sharon Kaasalainen, Cees M Hertogh, Sophie Pautex, Lieve Van den Block

**Affiliations:** ^1^ Research Centre on Aging University Institute of Geriatrics of Sherbrooke Sherbrooke, QC Canada; ^2^ Faculty of Medicine and Health Sciences Community Health Sciences University of Sherbrooke Sherbrooke, QC Canada; ^3^ Faculty of Medicine and Health Sciences Family Medicine University of Sherbrooke Sherbrooke, QC Canada; ^4^ Schulich School of Law Dalhousie University Halifax, NS Canada; ^5^ Faculty of Medicine Dalhousie University Halifax, NS Canada; ^6^ School of Nursing McMaster University Hamilton, ON Canada; ^7^ EMGO+ Institute for Health and Care Research General Practice & Elderly Care Medicine Vrije Universiteit Medical Center Amsterdam Netherlands; ^8^ Geneva University Hospital Community Medicine and Primary Care Geneva Switzerland; ^9^ Vrije Universiteit Brussel UGhent End-of-Life Care Research Group Brussels Belgium

**Keywords:** euthanasia, dementia, decisional incapacity, advance directive, attitude, survey, Canada

## Abstract

**Background:**

Alzheimer’s disease and related disorders affect a growing number of people worldwide. Quality of life is generally good in the early stages of these diseases. However, many individuals fear living through the advanced stages. Such fears are triggering requests for medical assistance in dying (MAiD) by patients with dementia. Legislation was recently passed in Canada and the province of Quebec allowing MAiD at the explicit request of a patient who meets a set of eligibility criteria, including competence. Some commentators have argued that MAiD should be accessible to incompetent patients as well, provided appropriate safeguards are in place. Governments of both Quebec and Canada are currently considering whether MAiD should be accessible through written requests made in advance of loss of capacity.

**Objective:**

Aimed at informing the societal debate on this sensitive issue, this study will compare stakeholders’ attitudes towards expanding MAiD to incompetent patients with dementia, the beliefs underlying stakeholders’ attitudes on this issue, and the value they attach to proposed safeguards. This paper describes the study protocol.

**Methods:**

Data will be collected via a questionnaire mailed to random samples of community-dwelling seniors, relatives of persons with dementia, physicians, and nurses, all residing in Quebec (targeted sample size of 385 per group). Participants will be recruited through the provincial health insurance database, Alzheimer Societies, and professional associations. Attitudes towards MAiD for incompetent patients with dementia will be elicited through clinical vignettes featuring a patient with Alzheimer’s disease for whom MAiD is considered towards the end of the disease trajectory. Vignettes specify the source of the request (from the patient through an advance request or from the patient’s substitute decision-maker), manifestations of suffering, and how close the patient is to death. Arguments for or against MAiD are used to elicit the beliefs underlying respondents’ attitudes.

**Results:**

The survey was launched in September 2016 and is still ongoing. At the time of submission, over 850 respondents have returned the questionnaire, mostly via mail.

**Conclusions:**

This study will be the first in Canada to directly compare views on MAiD for incompetent patients with dementia across key stakeholder groups. Our findings will contribute valuable data upon which to base further debate about whether MAiD should be accessible to incompetent patients with dementia, and if so, under what conditions.

## Introduction

Medicine aims to relieve patient suffering and cure illness [1]. When patients can no longer be cured, palliative care aims to improve quality of life by relieving suffering [2]. Palliative care has expanded over the past decades, although accessibility gaps remain, notably for patients with Alzheimer’s disease and related disorders whose illnesses are still too often not recognized as terminal [3,4]. Moreover, despite quality palliative care, some patients (with and without dementia) experience treatment-refractory symptoms that may lead to medical assistance in dying (MAiD) requests as a last resort to alleviate suffering [5,6]. Whether MAiD should be accessible to incompetent patients with dementia raises complex ethical and practical issues. We designed a study to investigate the views of stakeholders on these issues in Quebec, Canada. In this paper, we summarize current knowledge on these issues, state the objectives of our study, describe its methodology, and discuss its strengths and limitations.

### Legal Landscape on MAiD Internationally in Canada, and in Quebec

Outside of Canada, euthanasia and/or physician-assisted suicide have now been legalized in four countries (The Netherlands, Belgium, Luxemburg, and Colombia), four US states (Oregon, Washington, Vermont, and California) and the District of Columbia. In the scientific literature, *euthanasia* commonly refers to, “the administration of drugs with the explicit intention of ending the patient’s life at his or her explicit request” and *physician-assisted suicide* refers to, “the prescription or supply of drugs with the explicit intention of enabling the patient to end his or her own life” [7]. An *explicit request* can be made contemporaneously or previously (ie, in advance of incapacity). Physician-assisted suicide is also allowed by operation of a court decision in Montana. In Switzerland, euthanasia is illegal but assisted suicide (whether by a physician or nonphysician) is only prohibited when done for a “selfish motive” [8-11]. Between 0.1% and 4.6% of all deaths involve MAiD in countries where it is legal [12,13].

In most permissive jurisdictions, MAiD is not available to individuals with dementia. Patients are either still capable but not near enough to the end of life, or they are close enough to the end of life but no longer capable. However, in the Netherlands, where being at the end of life is not a condition of eligibility for MAiD, 109 euthanasia requests from competent patients in the early stages of dementia were granted in 2015 [6]. Furthermore, the Dutch legislation allows a physician to comply with a euthanasia request made by a formerly competent patient through an advance request, as long as all of the “criteria of due care” are met (notably including unbearable suffering). To date, four cases have been reported regarding Dutch patients who requested euthanasia while competent via a written request, and whose requests were granted after they had become incompetent [6,14]. Similarly, the law in Belgium does not require patients to be at the end of life or terminally ill, and does permit some access to MAiD through written requests made in advance of loss of capacity. However, in these cases the patient must be unconscious. In Luxembourg, a patient must be in a terminal condition, and access to MAiD is permitted through an advance request (provided the patient is unconscious). These requirements mean that many patients with dementia will not qualify for MAiD, but some will.

Until recently, MAiD was prohibited in Canada under several provisions of the *Criminal Code*. There have been court challenges to these provisions over the last 25 years, the most notable being *Rodriguez v. British Columbia (Attorney General)* in 1993 [15] and, more recently, *Carter v. Canada (Attorney General)* in 2015 [16]. In the first case, the Supreme Court of Canada dismissed the appeal of Sue Rodriguez, a woman living with amyotrophic lateral sclerosis who had challenged the validity of the *Criminal Code* prohibitions on MAiD [15]. This decision was overturned on February 6, 2015 by a unanimous decision of the Supreme Court in *Carter v. Canada* [16] *.* The judges ruled that the prohibitions violate section 7 of the *Canadian Charter of Rights and Freedoms*.

The Court’s ruling catalyzed the Government of Canada to engage in consultation and draft legislation specifying the eligibility criteria and procedural safeguards for access to MAiD. Bill C-14 came into force on June 17, 2016 [17], enacting exemptions from criminal liability for physicians and nurse practitioners who provide MAiD, and for others who assist them. Eligibility is restricted to a competent adult who makes a voluntary and well-considered request for MAiD and has a, “grievous and irremediable medical condition” [17]. Canada Bill C-14 defines *medical assistance in dying* as:

The administering by a medical practitioner or nurse practitioner of a substance to a person, at their request, that causes their death, or the prescribing or providing by a medical practitioner or nurse practitioner of a substance to a person, at their request, so that they may self-administer the substance and in doing so cause their own death [17]

MAiD thus encompasses both euthanasia and physician-assisted suicide, as defined above. A person has a grievous and irremediable medical condition only if all of the following criteria are met:

(a) they have a serious and incurable illness, disease, or disability; (b) they are in an advanced state of irreversible decline in capability; (c) that illness, disease, or disability or that state of decline causes them enduring physical or psychological suffering that is intolerable to them and that cannot be relieved under conditions that they consider acceptable; and (d) their natural death has become reasonably foreseeable, taking into account all of their medical circumstances, without a prognosis necessarily having been made as to the specific length of time that they have remaining [17]

Contrary to recommendations made by a Provincial-Territorial Expert Advisory Group on Physician-Assisted Dying [18] and a Special Joint Committee of the House and Senate on Physician-Assisted Dying [19], the federal legislation does not allow a person to access MAiD through a request made in advance of loss of capacity. However, the legislation mandated an independent review and reporting back to Parliament on several issues, including advance requests. The government has commissioned the Council of Canadian Academies to independently manage the review and report to Parliament by December 2018.

On June 10, 2014, eight months before the Supreme Court’s ruling in *Carter v. Canada*, the Quebec National Assembly adopted *An Act respecting end-of-life care* (Bill 52) which codifies recommendations made by the Quebec College of Physicians [20] and the province’s Select Committee on Dying with Dignity [21]. Briefly, Bill 52 first affirms the right of everyone to end-of-life care that is appropriate to their needs. The bill also regulates continuous palliative sedation, establishes an advance medical directives regime, and permits MAiD under strictly defined circumstances [22]. In Quebec Bill 52, MAiD is defined as:

Care consisting in the administration by a physician of medications or substances to an end-of-life patient, at the patient’s request, in order to relieve their suffering by hastening death [22]

This definition corresponds to euthanasia, as defined above. Nurse practitioners are not authorized to administer aid in dying under the Quebec legislation. The legislation became effective on December 10, 2015. Eligibility for MAiD is restricted to competent adults from Quebec who are at the end of their lives, have made persistent explicit requests for MAiD, and:

Suffer from a serious and incurable illness, are in an advanced state of irreversible decline in capacity, and experience constant and unbearable physical or psychological suffering which cannot be relieved in a matter the patient deems tolerable (article 26) [22].

For greater clarity, article 51 specifies that MAiD may not be requested by means of an advance medical directive.

Quebec Bill 52 has drawn both opposition and support, with some supporters recommending that access to MAiD be extended to incompetent patients, provided appropriate safeguards are in place [23]. Proposed safeguards for MAiD, should it be accessible to incompetent individuals, have included: an explicit request made in an advance directive by the patient while competent; consent from the patient’s legal representative; and prior authorization of a local or provincial body, where relatives and health professionals could be heard [24]. Ultimately, Quebec decided not to give incompetent patients access to MAiD through Bill 52, noting a lack of societal consensus on this issue [25]. However, recently the Minister of Health and Social Services announced that an expert group will be tasked with studying whether access to MAiD should be permitted through requests made in advance of loss of capacity. This initiative has obvious implications for patients with dementia.

In both Canada and the province of Quebec, eligibility criteria for MAiD currently exclude patients who have become incompetent due to Alzheimer’s disease or other forms of dementia. These are serious incurable conditions that progressively and irreversibly erode patients’ abilities to perform basic activities of daily living. Additionally, many affected individuals develop serious clinical complications (eg, eating problems, pneumonia) and distressing symptoms (eg, pain, dyspnea) that are difficult to manage [26-29]. However, by the time a patient satisfies these criteria, he or she is unlikely to be competent. Strong opinions have been voiced in support of, and in opposition to, the exclusion of patients with advanced Alzheimer’s disease and other forms of dementia. The issue of respecting MAiD requests that are to be carried out after the patient has lost capacity is a complex health care issue that brings diverse societal values and beliefs into relief and conflict; these are briefly reviewed below.

### Arguments For and Against MAiD, in General and for Incompetent Patients With Dementia

Arguments in favor of MAiD generally include individual autonomy and freedom of choice, the inability to relieve suffering in some cases, the absence of a moral distinction between withholding/withdrawing potentially life-sustaining treatment and MAiD, and the claim that permitting MAiD allows the establishment of stronger safeguards and oversight for the entire spectrum of end-of-life medical care through carefully-designed regimes. Arguments against MAiD include: the sanctity of life; the need to protect socially vulnerable populations from abuse and social discrimination; concerns about the *slippery slope* which (depending on the interpretation) could lead to more abuse, or to the legislation being extended to incompetent patients; the risk of impeding the development of palliative care; and ethical tensions faced by physicians who object to MAiD on moral grounds, but could feel or be obliged to carry out the request [11,30-33].

Other arguments are raised against MAiD when referring specifically to patients rendered incompetent by advanced dementia: patients’ potential to adapt to their disease, which may change previously expressed wishes (the so-called “disability paradox”); the impossibility of health care providers and families engaging in meaningful conversations with the patient to confirm the wish to die; and practical difficulties in assessing suffering, balancing current preferences against earlier wishes laid down in a now-forgotten request, and choosing the right moment to carry out the request. Complying with an advance request for MAiD also raises the philosophical question of whether a request made by a previously competent person should have any authority over the life of a person who now has severe dementia [34-44].

### Attitudes of Stakeholders Towards MAiD

Major groups of stakeholders likely to be impacted by extending MAiD to incompetent patients include older adults, relatives of patients with dementia, physicians, and nurses. Systematic reviews of quantitative studies from several countries, including Canada, show increasing support from these groups of stakeholders for MAiD in cases of competent terminally-ill patients experiencing severe pain who make an explicit request [10,11,45-47]. Far fewer studies have investigated opinions of stakeholders on MAiD for patients with dementia [48-58]. None of these studies were conducted in Canada and only two have focused exclusively on this issue [53,56]. As shown in Table 1, support was found to be higher in the general population and lower among physicians, with nurses’ opinions falling in between. Support for advance requests for MAiD is also stronger among the general public than among health care practitioners, who raise numerous issues regarding their use [37,39].

In conclusion, growing knowledge of possible clinical complications of advanced dementia, and current access to MAiD for competent adults, will likely trigger advance requests for MAiD from Canadians diagnosed with dementia [11,59,60]. To date, no study has investigated the attitudes and beliefs of Canadians on this complex and sensitive issue. Similarly, no study has examined whether Canadians support other end-of-life medical practices in advanced dementia, such as the withholding of antibiotics for a life-threatening pneumonia or continuous deep sedation for agitation refractory to treatment. This study will shed light on these specific issues, providing much-needed evidence to support future health care policy development on end-of-life care for Canadians with advanced dementia.

**Table 1:** Studies that have elicited attitudes towards assistance in dying for incompetent patients (mostly due to dementia).

First author (year of publication)		Country	Age of sample, years (standard deviation)	Sex of sample, male (%)	Response rate (%)	Sample size	Percent judging the practice (or its legalization) acceptable^a^
**General Public**							
	Koenig (1996) [49]	United States (Durham, NC)	61% over 75 years	23	86	168	14
	Van Holsteyn (1998) [50]	The Netherlands	18 or older	unspecified	46	911	45
	Ryynänen (2002) [51]	Finland	18 to 70	42	59	587	48
	Rietjens (2005) [52]	The Netherlands	20 to 93	39	78	1388	62
	Williams (2007) [53]	United Kingdom (London)	43 (17)	46	71	725	57 to 59
	Lindblad (2010) [54]	Sweden	20 to 84	50	52	625	60 to 65
	Kouwenhoven (2013) [55]	The Netherlands	53 (15)	54	78	1960	77
**Relatives of patients with dementia**							
	Rurup (2006) [56]	The Netherlands	57	38	72	136	89
**Physicians**							
	Ryynänen (2002) [51]	Finland	24 to 87	48	62	506	8
	Rietjens (2005) [52]	The Netherlands	63% aged 40 to 55	76	81	391	6
	Rurup (2006) [56]	The Netherlands	41	51	96	107	16
	Lindblad (2010) [54]	Sweden	32 to 79	69	56	667	13 to 15
	Kouwenhoven (2013) [55]	The Netherlands	51 (8)	65	41	793	33
**Nurses**							
	Ryynänen (2002) [51]	Finland	20 to 63	6	73	582	23
	Rurup (2006) [56]	The Netherlands	34	17	94	148	57
	Gielen (2009) [57]	Belgium (Flanders)	44 (9)	12	70.5	415	52 to 56
	Inghelbrecht (2009) [58]	Belgium (Flanders)	42% aged 36 to 45	12	62.5	3321	57
	Kouwenhoven (2013) [55]	The Netherlands	44 (11)	10	unspecified	1243	58

^a^Range reported when several items were used to measure acceptability

### Research Objectives and Hypotheses

Restricted to Quebec with plans for extension to other Canadian provinces, this study will elicit and compare the attitudes of four groups of stakeholders (seniors, relatives of persons with dementia, physicians, and nurses) towards MAiD for incompetent patients with dementia, the beliefs underpinning stakeholders’ positions on this matter, and their opinions as to whether proposed safeguards can adequately protect incompetent patients. Based on findings in other countries [11,31], we expect that: (1) support for MAiD for incompetent patients with dementia will be higher among seniors and relatives than among health care practitioners; (2) support among health care practitioners will increase with additional safeguards, without reaching the level of support found in the two other groups; (3) religiosity, *slippery slope*, autonomy, and dying-with-dignity arguments will affect respondents’ permissiveness toward MAiD for incompetent patients with dementia; and (4) the relative weight of these arguments in shaping opinions will vary across the four groups of stakeholders.

## Methods

### Study Design, Target Groups, and Sampling

An anonymous province-wide postal survey using clinical vignettes will be conducted on random samples of French-speaking Quebec residents belonging to one of four groups of stakeholders: (1) community-dwelling seniors aged 65 years or older, (2) relatives of persons with dementia, and (3) physicians and (4) nurses likely to be involved in end-of-life decision making. In Quebec, 94% of the population speaks French [61].

The random sample of community-dwelling seniors will come from the Quebec health insurance database. Relatives of patients with dementia will be reached through regional Alzheimer Societies. To protect their members’ right to privacy, participating Societies will randomly select a predetermined number of potential participants (proportional to the size of their memberships) and distribute the survey package directly to them per our instructions. Assistance in managing the survey will be provided by our research staff to any Society that expresses the need. Practicing physicians and nurses will be recruited through their respective professional bodies, excluding those in full-time administrative, teaching, or research positions due to their limited direct contact with patients. Specialties for physicians will be restricted to family medicine, geriatrics, internal medicine, neurology, psychiatry, and intensive care; for nurses, specialties will be restricted to geriatrics/gerontology and end-of-life care.

### Postal Survey

**Figure 1:**
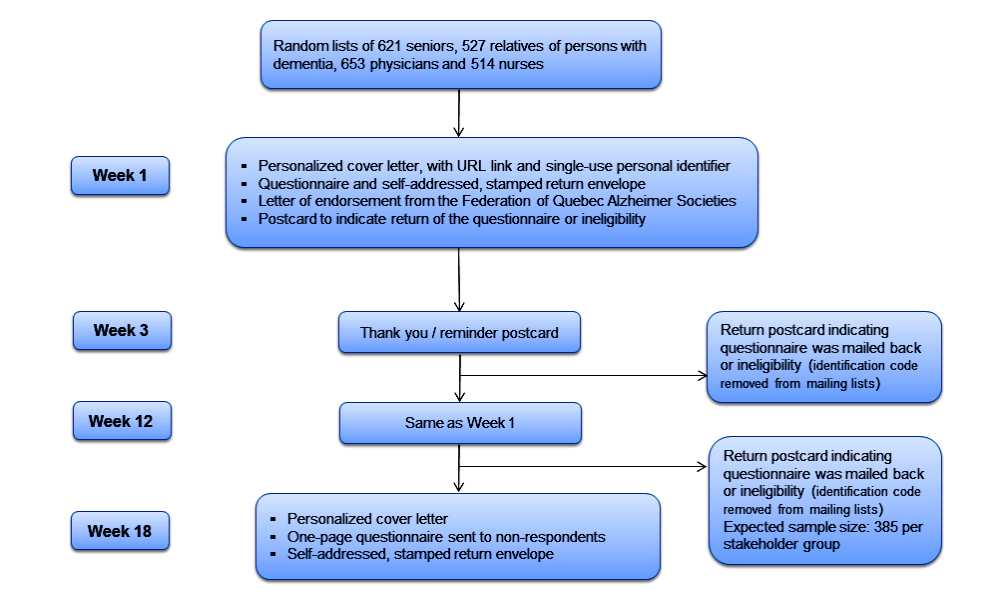
Outline of the postal survey.

The survey and questionnaires were designed using strategies shown to maximize response rates and data quality [62]. As depicted in Figure 1, randomly sampled individuals receive a personalized cover letter and accompanying materials in week 1, and a thank-you/reminder postcard in week 3. Nonrespondents are mailed a second survey package in week 12. The personal letter states the aim of the survey, explains how recipients were chosen, mentions that completing the questionnaire requires 20 minutes on average (based on pretesting), and addresses issues of privacy and anonymity. The letter also provides the Internet link (website address) to the online version of the questionnaire as well as the recipient’s single-use personal identifier. A self-addressed and stamped return envelope is enclosed in the survey package for those who prefer to complete the printed questionnaire.

A letter of endorsement from the Federation of Quebec Alzheimer Societies is also included, with a postcard bearing the respondent’s name, to be returned separately from the questionnaire. Returned postcards make it possible to identify sampled individuals who have returned the questionnaire–either by mail or electronically–while preserving the anonymity of their answers. The postcard also serves the purpose of identifying sampled individuals who are no longer eligible (eg, seniors who are now institutionalized or too cognitively impaired to participate). The names of those who return the postcard are immediately removed from the mailing list to prevent further reminders. At the close of the postal survey, nonrespondents receive a 3-item questionnaire asking: (1) for their reasons for not participating (eg, felt questions were biased, lack of time, or doubt that anonymity can truly be preserved); (2) how comfortable they are with the current Quebec legislation that gives competent patients access to MAiD if certain conditions are met; and (3) whether they favor or oppose allowing physicians to administer MAiD to incompetent patients, with proper safeguards in place. The latter two questions will be used to assess nonresponse bias.

### Questionnaires

After stating the eligibility criteria for MAiD as defined in Quebec’s *Act respecting end-of-life care*, the 3-part questionnaire presents a series of multiple-choice questions, with space for the respondent’s comments. Part 1 elicits attitudes towards MAiD and other end-of-life practices. Two sets of clinical vignettes are used for that purpose. The first vignette features a cancer patient who is eligible for MAiD. Using a 5-point Likert-type scale, respondents are asked to what extent they find it acceptable for a physician to sedate the patient continuously until death to relieve suffering, or to comply with the patient’s request for MAiD. The second set, containing 7 interrelated vignettes, features a woman moving along the dementia trajectory, from the early stage when she is diagnosed with Alzheimer’s disease to her final days of life. End-of-life practices (besides MAiD) for which support or opposition is investigated include withholding antibiotics for a life-threatening infection and continuous deep sedation for refractory agitation. Vignettes specify the source of the request for MAiD (an advance request made in writing by the patient while she was still competent, or her family), whether the patient appears to be suffering (eg, showing signs of distress, crying regularly), and whether death seems imminent. Vignettes are kept as nontechnical as possible to be easily understood, regardless of the respondent’s medical knowledge. A sensitive and neutral tone is used throughout the questionnaire to prevent response bias. Part 1 ends with a list of statements designed to capture respondents’ reasons for supporting or opposing MAiD, generally and for incompetent patients in particular. Reasons include, for example: religious and moral objections, respect for patient autonomy, practical difficulties in ascertaining whether an incompetent patient is suffering unbearably, and concerns about the *slippery slope*.

Part 2 explores related issues, such as whether respondents have filled out an advance directive for themselves, personally know someone with dementia, or have ever accompanied a dying relative or friend. Respondents are also asked the likelihood that they would request MAiD in advance of loss of capacity, should they be diagnosed with Alzheimer’s disease, or ask a physician to comply with such a request drafted by a loved one under similar circumstances.

Part 3 collects sociodemographic data from all respondents (eg, age, gender, ethnicity, degree of religiosity) and contains additional group-specific questions. For seniors, these questions include educational attainment and self-rated health. Relatives are asked how long ago the person with dementia was diagnosed and their current level of cognitive functioning. Questions for physicians and nurses explore their experience in caring for dying and dementia patients, training in palliative care, and exposure to MAiD requests from patients or patients’ relatives. Physicians are also asked whether they would be willing to provide such assistance, were it legal. Few physicians currently administer MAiD in Quebec, and to preserve their anonymity, surveyed physicians are not asked whether they have in fact provided such assistance in the past.

Questionnaires were developed in English with input from renowned English-speaking content experts from countries where assisted dying is legal or not criminalized. The questionnaires were then translated into French and pretested through cognitive interviews performed by a research assistant with representatives of the four groups of stakeholders (n=20). Interviews were aimed at assessing the length of the questionnaires, clarity of the questions, uniformity in comprehension, and respondent comfort with the content [63]. Following these interviews, minor modifications were made to some questions, which aimed at emphasizing differences between vignettes (eg, advanced vs. terminal stage of Alzheimer’s disease, presence vs. absence of a written request). Questionnaires were then converted to a Web format. The Web questionnaires were developed using the latest version of LimeSurvey [64], a free open-source online survey application that allows assigning a single-use password to each sampled individual. The server hosting the LimeSurvey software uses proven encryption methods (Transport Layer Security) to transmit survey answers to a secure server and export collected data into a statistical package for analysis. Before launching the survey, the Web version was tested in-house and with several remote participants on different operating systems, browsers, and platforms, and for different types and speeds of Internet access. Systematic troubleshooting was performed to uncover unforeseen technical problems.

### Data Analyses

The data will be analyzed in four consecutive steps. First, we will compare nonrespondents, respondents to the one-page questionnaire only, and late versus early respondents to the full questionnaire, to detect response bias and establish sample weights where needed. Response rates will be reported as recommended by the American Association for Public Opinion Research. In Step 2, we will study patterns of item nonresponse and determine whether imputing missing data would be appropriate [65]. Next, we will summarize participant answers to the questionnaire, and compare distributions of answers within respondents, as well as within and between the four groups of stakeholders. Within-respondent comparisons will require multilevel analyses, since answers to different questionnaire items will be correlated [66]. For instance, the proportions of respondents who support MAiD at the advanced versus terminal stages are not independent and hence cannot be compared using the usual Chi-square test. Estimations of model parameters will be based on maximum likelihood with adaptive quadrature, which outperforms other methods in terms of bias and efficiency of the estimates when the number of study participants is large, as is typical of survey research [67]. Respondent characteristics will subsequently be added to the models in a stepwise fashion to identify additional correlates of response patterns in addition to group membership. Residual analyses will be conducted to assess the tenability of the assumptions underlying the statistical models, and to identify influential observations and outliers. Multilevel modelling will be conducted with SAS Proc NLMIXED [68], which offers a wide choice of integral approximations and optimization techniques.

### Sample Size

The data from our four samples will first be summarized with proportions and associated confidence intervals. In the worst-case scenario of equal proportions for and against a given end-of-life practice, two-sided 95% confidence intervals for proportions require 385 respondents per sample when the semiinterval width is set at 5% (nQuery Advisor, version 7.0). The sample size required for reliably fitting multilevel models depends on several factors, including sample size at each level of the analysis, number and type of predictors included in the model, intraclass correlation, and model complexity. Recent Monte Carlo simulation studies on multilevel models for binary and continuous outcomes suggest that 100 to 200 level-2 units (ie, survey respondents) with 5 to 10 level-1 units (ie, questionnaire items) ensure model convergence and provide adequate power for testing fixed and random effects [69]. To determine the number of questionnaires to mail out to achieve the target of 385 respondents per group, we applied response rates derived by averaging those reported in Table 1 with our own [70,71]. The resulting numbers are: 621 seniors, 527 relatives of persons with dementia, 653 physicians, and 514 nurses, for a total of 2315 potential participants.

### Ethical Considerations

This study will investigate views on sensitive issues. While there are no physical risks to participants, psychological risks must be acknowledged. Questionnaires may trigger emotional distress in some participants or revive painful memories of the death and suffering of a loved one. In an effort to minimize these risks, the cover letter that accompanies the questionnaire includes contact details for a support resource, if needed. Participation in the survey is voluntary and answers are anonymous. Signed consent is not required; in anonymous surveys, implicit consent is inferred from respondents who return the questionnaire [72]. All information needed for informed consent is provided in the cover letters, including a toll-free telephone number and email address for those who have questions or concerns about the survey. Personal information on sampled individuals is coded, and access to password-protected lists of codes is restricted to the research team. Sampling lists will be destroyed five years after the end of the study. The Research Ethics Board of the University Institute of Geriatrics of Sherbrooke granted ethical approval of the survey design and questionnaires (file # 2016-623).

## Results

The survey was launched in September 2016 among relatives of patients with dementia, physicians, and nurses, and is still ongoing. The survey will be launched among older adults as soon as we receive a random list of names extracted from the Quebec health insurance database.

## Discussion

To the best of our knowledge, this study will be the first to uncover Quebec stakeholders’ attitudes towards MAiD for incompetent patients with dementia, which is a vulnerable and rapidly expanding population of patients. Dementia affects more than 37 million people worldwide, with a projected increase to over 115 million by 2050 [73]. The Alzheimer Society of Canada has estimated that 564,000 Canadians were living with dementia in 2016, and this number is expected to rise to 937,000 by 2031, representing an increase of 66% [74]. Life expectancy after a dementia diagnosis is believed to lie between 3 and 12 years [75]. Because no cure is foreseen in the near future, many people will die with or from dementia. Over the last decade, deaths attributed to Alzheimer’s disease rose by 39% in the United States [76]. Although quality of life can be good in the early stages, dementia still ranks among the most feared clinical conditions in modern societies [77]. Indeed, some perceive this syndrome as a “fate worse than death” and dread the prospect of living through the advanced stages of dementia [78,79]. People are apprehensive about the progressive loss of decisional capacity and control, prolonged dependence upon others for their most basic needs, inability to report physical and psychological suffering, and lengthy periods of institutionalization before death. As the prevalence of dementia continues to rise, a growing number of individuals who do not want to experience the full course of dementia might request MAiD.

Do stakeholders believe that MAiD should be made available to patients who have reached an advanced stage of dementia? Under what circumstances? Are other end-of-life practices viewed as more appropriate? Can proposed safeguards on MAiD adequately protect vulnerable individuals from abuse and coercion? Do views on these issues vary markedly within and between groups of stakeholders? By using a proven research methodology and identical questions across stakeholder groups, this study will provide answers to these questions, which have yet to be explored in Canada, and have only been partially investigated in other countries.

### Strengths and Limitations

The current study has strengths and limitations. Strengths include: the timeliness of the survey, which will inform ongoing legislative activities; random selection of potential respondents from four highly relevant groups of stakeholders; the anonymity of answers, which decreases bias due to social desirability; the care taken in designing and testing the questionnaires with input from international content experts; and our decision to administer a common set of questions to all four groups of stakeholders, enabling direct comparison of their views on MAiD for incompetent patients with dementia. The presence of investigators on the research team who support, and others who oppose, extending MAiD to these patients is another strength, as it minimizes the risk of biased questions and increases uptake of research findings [80].

Although not without limitations, surveys contribute invaluable data to inform ethical debates, public policy development, and future research on sensitive issues such as whether MAiD should be extended to incompetent patients with dementia [81]. Postal surveys offer many advantages over other data collection methods. First, such surveys are relatively inexpensive for surveying large and geographically dispersed populations, provide greater flexibility for the respondents, maintain anonymity, and yield higher response rates than telephone surveys [62]. In our survey, sampled individuals have the option to complete a paper or online version of the questionnaire, which is a strategy shown to yield even higher response rates [82,83]. Second, earlier studies conducted abroad provide a solid basis for the design of high-quality clinical vignettes featuring incompetent patients, MAiD requests, and end-of-life practices [49-58]. Additionally, the practical problems and moral dilemmas created by advanced MAiD requests, and the arguments for and against MAiD for incompetent patients with dementia, have been thoroughly reviewed [34-44]. These reviews have provided ample materials from which to formulate questionnaire items for exploring beliefs underlying attitudes.

Low response rates threaten the external validity of findings from attitude surveys. To counter this problem, both the survey and the questionnaires were designed using strategies that comprehensive reviews have shown to be effective [84,85]. Response rate is an imperfect indicator of survey quality, however. Empirical assessments over the past decade have concluded that response rates may not be as strongly associated with survey quality as was generally believed [86]. The degree to which respondents differ from the target population is the central issue. Well-recognized approaches to assess nonresponse bias and minimize its effect are part of our analytical plan and include: inviting initial nonrespondents to complete a shorter questionnaire with only key measures of interest, comparing respondents with nonrespondents using information available in the sampling frame, comparing early versus late respondents on personal characteristics and answers to survey questions, and weighting analyses of the variables of primary interest.

One limitation is the restriction of the survey to the province of Quebec. We plan to extend the survey to the rest of Canada in the near future, using the same questionnaires to enable provincial/territorial comparisons. Short case descriptions with a limited number of possible answers fall short of capturing the complexity of end-of-life decision making [52]. Our upcoming pan-Canadian survey will include a qualitative component aimed at gaining deeper insight into respondents’ thought processes and the reasons behind their support for, or opposition to, MAiD for incompetent patients with dementia [43]. Opinions are elicited using specific vignettes. Whether opinions extend to other clinical contexts involving MAiD requests from incompetent patients with dementia will remain unknown. We chose not to elicit attitudes towards extending MAiD to patients at earlier stages of dementia who are still competent. Including cases of early and late stage dementia in the same questionnaire would increase its length and possibly lower response rates. We also chose not to recruit in long-term care facilities, where most residents would be too cognitively impaired to provide reliable and valid answers to the survey questionnaire. We do not purposefully target seniors with dementia, even though those at an early stage of the disease would likely have the cognitive abilities to participate in the survey. We felt that a self-administered questionnaire was not ethically appropriate for this subpopulation, given the sensitive nature of the subject under investigation [81]. However, as the views and concerns of this population regarding end-of-life practices in advanced dementia are highly relevant yet currently unknown, we are simultaneously conducting a qualitative study in this population. The data from persons with early dementia will be collected during face-to-face interviews, allowing the interviewer to respond promptly should questions trigger negative emotions in some participants. Combining findings from our survey with those from the in-depth interviews will allow more nuanced recommendations as to whether MAiD should be expanded to incompetent patients with dementia.

## Abbreviations

MAiD: medical assistance in dying
